# Supplementary material for: Are carbon emissions trading and green financial instruments synergistic? -Comprehensive quantitative research based on content analysis
Source: PLoS One. 2024 Mar 7;19(3):e0298601. doi: 10.1371/journal.pone.0298601 (PMC10919601; doi:10.1371/journal.pone.0298601)
Supplement: S1 Data — (ZIP) [file pone.0298601.s001.zip › Data/地级市Z_F工作报告.zip!/╘╞─╧/╟·╛╕╩╨/└┤╫╘╣1⁄2╓┌║┼í╛┬φ┐╦╩2╛▌═°í┐_2015.docx]

各位代表：

我代表市人民政府，向大会报告工作，请予审议，并请市政协委员提出意见。

一、2014年工作回顾

刚刚过去的2014年，是我市经济社会发展形势严峻复杂、困难挑战异常突出的一年，也是重大基础设施建设、煤炭产业转型升级、“四城”联创、重点领域改革等工作迈出坚实步伐的一年。在市委的领导下，市人民政府认真贯彻落实党中央、国务院和省委、省政府及市委的决策部署，紧紧抓住经济社会发展的重点领域和关键环节，统筹抓好稳增长、调结构、促改革、惠民生和抗震救灾等重点工作，完成市内生产总值1649.4亿元，同比增长4%；规模以上固定资产投资1164.5亿元，同比增长14.1%；一般公共预算收入115.7亿元，同比下降4.8%；社会消费品零售总额427.7亿元，同比增长13.1%；外贸进出口总额4.5亿美元，同比增长20.4%；城镇和农村常住居民人均可支配收入25023元、8514元，同比分别增长9.1%、13.1%；单位生产总值能耗下降3.4%，城镇化率达42.6%；城镇登记失业率、人口自然增长率、居民消费价格总水平涨幅均控制在年初计划目标内。

（一）稳增长措施扎实有效。发挥政策稳增长的激励作用，狠抓中央、省稳增长系列政策措施的落实，出台了做好当前经济工作的36条、促进工业经济平稳增长的21条等系列政策措施，落实重点工业产品促销、电价补贴、纳规奖励资金3.2亿元，运用转贷应急资金为工业企业争取银行贷款12亿元，落实税收优惠政策为企业减负6.8亿元，新增银行贷款119.4亿元，直接融资66.3亿元。发挥投资稳增长的关键作用，建立市级领导挂钩联系重点项目包保责任制，市级安排项目前期工作经费6000万元，争取中央和省项目补助资金102.9 亿元，项目新开工1693个、在建2217个、竣工投产1685个。发挥招商引资稳增长的支撑作用，围绕重点产业实施定向招商、精准招商，市级签约项目达71个，协议总投资1028亿元；引进市外国内资金489.7亿元，同比增长28.2%；实际利用外资1.1亿美元，同比增长83.5%。发挥改革稳增长的推动作用，集中清理行政审批事项，加快网上服务大厅建设，在全省率先成立市级中介机构管理服务中心；以“2号章”的方式将152项行政审批事项下放到马龙现代产业园区管委会；推进商事制度改革，新增各类市场主体3万户，同比增长16.9%。

（二）产业转型升级积极推进。按照稳住存量和做大增量的思路，制定了产业转型升级实施意见，完成全市工业发展及布局规划，完成技改投资228亿元。深刻汲取“4·07”、“4·21”煤矿事故教训，顶住转型升级带来的经济下行压力，按照“三个一批”思路加快煤炭产业转型升级，完成132对地方煤矿年度关闭任务，占全省已关闭矿井的46.3%。非煤工业增加值达341.2亿元，同比增长11.8%；非公经济增加值占GDP的46.4%。高原特色农业发展取得新成效，落实强农惠农补贴和项目扶持资金13.3亿元，粮食总产达328万吨。引进联想控股佳沃集团、云南恒冠泰达等知名农业企业，新增市级以上农业龙头企业21户、精品庄园15个、农民专业合作示范社63个。建成13个部省级农业标准化示范区。投入牛源示范基地建设资金18.9亿元，推进54个“百千万”畜牧养殖示范场建设，实现畜牧业产值280.9亿元，同比增长8.8%。收购烟叶371.1万担，烟农收入达58.9亿元，实现烟叶税11.8亿元。土地承包经营权和林权流转面积分别达70万亩、20.7万亩。现代服务业发展步伐加快，编制现代服务业发展规划，明确发展重点。麒麟小坡、驰宏物流配送中心等综合物流园区建设稳步推进。完成乡村旅游发展规划，会泽大海草山获得国家4A级旅游景区称号。推进电子商务网站建设，“淘宝特色中国·曲靖馆”即将上线。金融服务业稳步发展，全市共有各类金融企业134家，从业人员达3万余人，金融机构各项存贷款余额分别达1702.3亿元、1137亿元，金融产业增加值占GDP的4.3%，被省金融办授予滇东次区域金融服务中心。科技创新扎实推进，建成省级重点实验室、院士工作站、专家工作站6个，新认定国家级高新技术企业18户、省级创新型试点企业和企业技术中心8户，新增国家地理标志商标4件、省著名商标25件。

（三）基础设施建设快速推进。开展综合交通“三年攻坚”行动，交通建设投资达101.1亿元，其中高速公路投资82亿元、在建里程402.1公里，是全省高速公路投资规模最大、在建里程最长的州市。宣曲、东过境高速公路顺利开工，江召、沾会、三宝至杨林、富源至兴义高速公路前期工作有序推进。沪昆客运专线转入路基、轨道铺筑阶段，启动高铁片区规划建设和曲靖至弥勒铁路、西南航空物流基地前期工作，宣威民航支线机场列入国家规划。启动农村公路“三年攻坚”行动，新建、改建农村公路2366公里。水利投资达36.2亿元，完成8件骨干水源、291件病险水库除险加固和8件中小河流治理工程。阿岗水库已获国家立项批准，并完成“四通一平”、管理用房建设和土地、实物指标调查工作；车马碧水库进入国家发改委项目建议书评估阶段，并启动了“四通一平”和管理用房建设。加大工业园区基础设施投入，落实省市补助资金1.5亿元，筹集园区建设资金10亿元，收储园区土地1.35万亩；启动马龙现代产业园区规划建设。能源和信息基础设施建设扎实推进，新增110千伏以上输电线路99.9公里，改造城网132公里、农网1683公里。建成中缅天然气管道门站和67公里城市天然气管道，昭通支线（曲靖段）、陆良支线前期工作进展顺利。启动“两化融合”示范试点工作，实现了4G网络县城全覆盖。

（四）城乡统筹取得新成绩。珠江源大城市总规修编、南盘江沿线区域规划、寥廓山风景名胜区规划、空间体系规划和市政系统综合规划取得阶段性成果，完成珠江源大城市2780公里地下管线普查探测、1450平方公里地形图补测更新。被列为全国新型城镇化综合试点市，国家卫生城市创建进入拟命名公示名单，国家园林城市创建通过省级考核验收。“两江”治理目标基本实现，打通中心城区8条21.4公里断头路，20.7公里的南小线建成通车，建筑立面改造、道路修复和拆除违章临时建筑等工作进展顺利。完成20个省级特色小城镇规划，有序推进11个市级重点小城镇建设。争取国开行贷款和直接融资175亿元，保障性住房及城市棚户区改造项目开工2.48万套、建成9338套。实施传统村落和历史文化名村保护，开展20个乡村清洁工程试点，改造农村危房1.4万户。发放农户小粮仓10万套。建成“五小”水利工程4.5万件，解决了45.6万人的饮水安全问题。启动三年城乡绿化攻坚行动，完成造林67.7万亩。超额完成四项主要污染物减排任务，23条重点河流水质断面监测达标率为90%，17个县级以上饮用水源地水质全面达标。《云南省曲靖城市管理条例（修订）》经省人大常委会审议通过。实施14个“整乡推进”、53个行政村和58个自然村“整村推进”项目，实施扶贫安居工程2600户，易地搬迁2100人，惠及24.6万贫困群众。

（五）民生事业发展成效明显。落实新建标准化幼儿园和县城中小学补助政策，市民中迁建、曲一中卓立学校开工建设，曲一中新校区、市二中改扩建前期工作有序推进，新建成各类校舍79.2万平方米。出台优秀骨干教师市政府特殊津贴制度。市中医院、市妇幼保健院建设加快推进，新建、改扩建乡镇卫生院23个、村（社区）卫生所（室）124个，医疗联合体改革取得实效。发放各类创业贷款11.5亿元，扶持1.6万人创业、带动4.3万人就业，新增城镇就业3.4万人。各项社会保险参保人数累计达492.5万人，在全省率先实施城乡居民大病补充保险。新农合减免费用16.5亿元。落实困难群众各类救助政策，为50.9万人发放城乡低保金8.5亿元。高质量完成曲靖文化体育公园建设，成功承办第十四届省运会、第十届省残运会。建成49个乡镇文化站、社区文化活动室。被列为全国养老服务业综合改革试点地区，新建成7个农村敬老院、41个社区居家养老服务中心。启动实施“单独两孩”政策。民族团结进步边疆繁荣稳定示范乡、村建设稳步推进。会泽地震灾区抗震救灾工作有力、有序、有效开展，完成灾后重建规划编制，争取国家、省补助和社会捐赠资金36亿元，恢复重建工作全面展开。

（六）社会治理能力不断提升。建立“党政同责、一岗双责、齐抓共管”的安全生产责任体系，落实安全生产企业主体、部门监管和属地管理“三个责任”，探索安全生产监管职责清单、失信惩戒机制和第三方安全监管机制，健全驻矿监督员管理、煤矿安全隐患举报奖励等制度。坚持常态化安全生产隐患排查治理，高密度、全覆盖抓好煤矿、非煤矿山、道路交通、消防等重点领域的安全隐患排查整治。食品药品三级监管机构和四级监管网络基本建立，强化从生产加工到流通消费的全过程监管。健全治安防控“三张网”，立体化社会治安防控体系不断完善。开展领导干部“大接访、大下访”和化解信访积案“百日会战”活动，“大调解”工作体系进一步完善，一批信访突出问题得到有效化解。

（七）政府自身建设得到加强。扎实开展党的群众路线教育实践活动，公开曝光违反中央八项规定精神案件177次615人，政纪处分249人，问责干部294人。加强公共资金审计，查出违纪违规资金2.7亿元。全面清理超标办公室和公务用车，减少和规范公务出国，政府机关作风进一步好转。集中办结审批及服务事项244.3万件，公共资源交易总额达88亿元。工商、质监行政管理体制和食品药品监管体制进一步理顺，全面完成事业单位分类工作。认真办理人大代表议案、建议和政协委员提案。做好普法教育和法律援助等工作，“法治曲靖”建设迈出新步伐。

广播电视、妇女儿童、红十字、慈善、残疾人等事业，国防动员、双拥、国有资产监管、移民开发、档案、地方志、统计调查、社会科学、外事侨务、保密、地震、人防、气象、水文、邮政等工作均取得新成绩。

各位代表，过去的一年，我市在宏观经济下行压力不断加大、煤炭产业转型升级力度前所未有、增长动力转换举步维艰的大背景下，取得这样的成绩实属不易。这是省委、省政府和市委科学决策、正确领导的结果，是市人大、市政协有效监督、大力支持的结果，是全市上下同心同德、团结奋斗的结果。在此，我代表市人民政府，向全市广大干部群众，向人大代表、政协委员、各民主党派、工商联和无党派人士、各人民团体、社会各界人士，向驻曲部队指战员、武警官兵和中央、省属单位，向所有关心支持曲靖发展的同志们、朋友们，表示崇高的敬意和衷心的感谢！

在肯定成绩的同时，我们也清醒地看到，全市生产总值、一般公共预算收入、规模以上固定资产投资增速低于年初确定的目标，这既受宏观经济下行、投资增长乏力、房地产市场调整、煤矿停产整顿（整改）等诸多因素的影响，也与我们对新常态下的经济形势把握不准、工作措施不到位有关。

综合判断，今年我们仍然面临不少困难和挑战：产业层次低、发展方式粗放、创新驱动不足，新的经济增长点尚未形成。工业品市场萎缩，工业增长乏力。产业转型升级阵痛显现，经济、民生、生态、稳定等“立体性”困扰十分突出。基础设施和民生事业欠账较多。安全生产监管点多面广，基础仍不牢固。一些政府部门和工作人员依法行政和服务意识淡薄，为官不为、选择性作为、懒政怠政，一些领域消极腐败现象时有发生。对此，我们将采取更有针对性、实效性的措施，切实加以解决。

二、2015年工作安排

2015年，是全面深化改革的关键之年，也是“十二五”收官、“十三五”起步之年。国际环境复杂多变，国内“三期叠加”使经济发展形势更加严峻，但我们有信心、有条件、有能力克服困难、应对挑战。当前，我们仍处于大有作为的重要战略机遇期，“一带一路”、长江经济带、滇中城市经济圈战略将深入推进，国家将继续实施加快西部地区发展的一系列扶持政策，全面深化改革将不断释放新活力，工业化、城镇化加速发展将催生巨大需求。同时，煤炭产业转型升级步伐加快，将为工业发展营造稳定的增长预期；综合交通、水利等基础设施投资将保持稳定的增长势头。只要我们科学认识、主动适应和引领经济发展新常态，趋利避害、顺势而为，充分发挥市场和政府“两个作用”，加快形成发展的“双引擎”，就一定能够化危为机，把曲靖的发展不断推向新高度、实现新跨越！

政府工作的总体要求是：全面贯彻党的十八大、十八届三中、四中全会、中央经济工作会议和省市委经济工作会议精神，以邓小平理论、“三个代表”重要思想、科学发展观为指导，深入贯彻落实习近平总书记系列重要讲话和对云南工作的重要指示精神，坚持稳中求进总基调，以提高经济发展质量和效益为中心，科学认识、主动适应和引领经济发展新常态，主动服务和融入“一带一路”、长江经济带、滇中城市经济圈一体化发展战略，积极推进滇中产业聚集区（新区）马龙现代产业园区、昆曲绿色经济示范带、麒沾马同城化、滇东次区域金融服务中心建设，抓项目增投资，抓转型调结构，抓改革促开放，抓实事惠民生，抓生态优环境，抓法治保稳定，抓安全促发展，抓党建强保障，确保实现全年经济社会发展目标。

经济社会发展的主要预期目标建议为：市内生产总值增长9%，一般公共预算收入增长7%，城镇常住居民人均可支配收入增长10%，农村常住居民人均可支配收入增长11%，居民消费价格总水平涨幅控制在3%以内。

重点抓好9项工作：

（一）抓项目增投资，不断增强发展新优势

发挥投资稳增长的关键作用，强化规划、用地、环保等审批服务，以路网、航空网、水网、能源网、互联网“五网”建设为重点，落实好“储备一批、开工一批、竣工（投产）一批”的良性滚动机制，加快62个省级、400个市级重点项目建设，力争规模以上固定资产投资突破1400亿元，增长20%。

加快路网、航空网建设。实施综合交通“三年攻坚”，谋划“五年会战”，完成市域综合交通体系规划，启动环市高速公路规划建设，推动市域内县际间高速公路的互联互通。确保南北大通道会泽段、普宣、西石高速公路建成通车，加快宣曲、东过境高速公路建设，开工建设江召、沾会高速公路，力争完成三宝至杨林、富源至兴义、陆良至泸西高速公路前期工作。支持沪昆客运专线曲靖段加快建设，启动曲靖北站建设，争取高铁经济区开工建设。推进曲靖至弥勒铁路、西南航空物流基地和宣威机场项目前期工作。加快国道213线江底至钢厂改造工程建设，启动国道326线杉木箐至宣威、省道会泽至者海公路建设，完成国道320线改造提升项目前期工作。力争完成交通投资150亿元。

加快水网建设。推进在建的18件骨干水源工程、103件小（二）型病险水库除险加固工程建设，新开工麒麟龙潭河水库等6件骨干水源工程、陆良平台子等237件小（二）型病险水库除险加固工程，争取阿岗水库、车马碧水库开工建设，开展沾益洞上河等12条中小河流治理。力争完成水利基础设施投资40亿元。

加快能源和互联网建设。强化协调服务，加快云南电网与南网主网鲁西背靠背直流异步联网项目建设，推进110千伏以上输变电工程建设，新增35千伏以上线路60公里，新增、改造10千伏线路640公里。推进17座压缩和液化天然气加气站建设，建成中缅天然气管道昭通支线（曲靖段）、陆良支线和 111公里城市天然气管道。加快页岩气区块勘探开发。推进宽带网络普及提速、“三网”融合和4G业务发展，推动金融IC卡多功能应用。力争完成能源及信息基础设施投资50亿元。

加快完善园区基础设施。坚持“先筑巢、后引凤”理念，再筹集10亿元资金，运用PPP等投融资模式，加快园区水、电、路、信息等基础设施建设。盘活土地资源，确保每个工业园区存量用地保持在1000亩以上。加快马龙现代产业园区建设，实现基础设施建设和项目落地新突破。

（二）抓产业转型升级，不断壮大经济实力

树立产业强则曲靖强的理念，加快培育一批新的经济增长点和增长极。

稳住存量做大增量。全面完成35对煤矿关闭整合任务，加快煤矿整合重组和改造升级步伐，促进煤炭产业健康发展。下决心解决产业结构单一、产业链短、附加值低等问题，推进众一化工煤焦油精深加工循环示范工程、华睿20亿方中泥煤制气等8个煤化工项目建设，加快发展以精细化工、合成材料、清洁能源、纺织原料等产业链为主的新型煤化工。加快不锈钢、镍合金、电解铝等产业的下游配套，构建从原材料到制成品的全产业链。鼓励企业技术创新，推动装备制造业由传统向现代、由制造零配件向整机生产转变。推进乔治白二期、东来丝绸1200万米绸缎等41个轻工项目建设，做强做大服装产业。抓好资源综合利用，发展新型墙材、预制水泥构件、特种玻璃、陶瓷制品和石材加工等产业。做强肉制品及火腿品牌，推动小黄姜、魔芋、银杏、万寿菊等产业走精深加工路子，壮大生物及食品加工产业。加快中宣液态金属等13个在建、红石岩风电等6个新开工项目建设，推动新能源新材料产业规模化发展。力争规模以上工业企业达580户，工业增加值突破790亿元，增长10%。

打造现代服务业发展新引擎。从规划、政策、要素配置等方面入手，推进生产性与生活性服务业协同发展。编制养老服务业发展规划，抓好全国养老服务业综合改革试点，鼓励发展一批民办养老机构，加快推进健康城项目。加大商贸流通企业培育、大型批发市场改造和乡镇农集贸市场建设，抓好10个商贸聚集区建设。加快麒麟小坡、罗汉山和马龙等综合物流园区建设，大力发展第三方物流。鼓励发展电子商务、服务外包等新兴服务业态，推进智慧城、“村邮户箱”等项目建设。启动滇东次区域金融服务中心建设，抓好金融要素交易平台建设，大力发展小微金融、互联网金融、资本管理、产业投资基金等多种金融业态，落实金融支持实体经济各项政策措施，确保信贷增量、直接融资规模超过上年水平。加强金融市场监管，规范民间融资行为，努力营造良好的金融生态环境。

积极扩大消费需求。多渠道增加城乡居民收入，提升消费能力，扩大住房、旅游、养老健康等领域消费。稳定住房消费，鼓励发展园区地产、商业地产、文化旅游地产和养老地产。落实职工带薪休假制度，促进旅游与城市、文化、生态和产业融合，加大重点旅游景区、景点和精品线路的包装、提升、推介力度，谋划推进十大旅游项目建设。力争社会消费品零售总额突破480亿元，增长13%。

优化产业布局。实施园区提升工程，完善园区规划，按照园区确定的主导产业，完善产业项目信息共享、能耗分担、利益分享机制，综合运用土地收储、节能减排和基础设施扶持资金等政策杠杆，引导各类园区围绕主导产业延伸产业链，推动工业发展园区化。统筹用好结构调整、技术创新、龙头企业扶持等财政专项资金，建立“政府资金引导、民间资本参股”的产业发展投融资平台，促进全市产业转型升级。

加强经济运行监测和要素保障。完善经济预测预警部门联席会议制度，实行月通报调度、季安排部署，提高经济工作的精细化、精准化水平。继续实行“一包五帮”和纳规奖励、优先采购本地工业产品等政策，积极争取扩大企业直购电交易范围。

（三）抓“三农”促增收，加快现代农业发展步伐

以保障粮食安全和促进农民增收为核心，进一步加强“三农”工作。

大力发展高原特色农业。稳定粮食播种面积在1000万亩以上，强化科技增粮、样板示范、高产创建等措施，巩固滇东粮仓地位不动摇。推动资源与资本、基地与龙头融合，巩固提升蔬菜、蚕桑、生姜、中药材、魔芋、核桃等特色产业规模，发挥联想佳沃蓝莓全产业链、康恩贝银杏庄园、褚橙庄园马龙万亩苹果示范项目的引领作用，推动农业内部三次产业融合互动，打造曲靖特色农产品品牌。大力培育发展各类新型农业经营主体，推进农产品加工龙头企业、农业精品庄园、农民专业合作示范社和部省级示范点建设。加快畜牧“百千万”示范场和牛源体系建设，配合农业厅抓好山地牧业科技示范园建设。提高淡水渔业发展规模和质量。推进现代烟草农业建设，促进烟叶生产提质增效。

加快美丽乡村建设。编制美丽乡村建设规划和特色民居图册，开展村庄布点规划，实施传统村落保护，加强农村建房规划管理和服务指导。加快美丽乡村示范点建设，留住乡愁记忆、传承地域文化。推进乡村垃圾清运和无害化集中处理，实施200个自然村乡村清洁工程。改造农村危房6万户。推进农村公路“三年攻坚”行动，新建改建农村公路2000公里以上。加快农村电网升级改造。完成3万件“五小”水利工程建设，解决42万农村人口饮水安全问题。

扎实推进精准扶贫。实施片区扶贫与精准扶贫“两轮”驱动，以整乡、整村推进综合扶贫为抓手，以专项扶贫、行业扶贫、社会扶贫为支撑，实施好省级5个整乡推进和43个行政村、50个自然村整村推进扶贫攻坚，完成易地搬迁1800人、扶贫安居工程2400户，减少贫困人口20万人。

继续深化农村改革。推进农村土地承包经营权确权登记，加快县乡土地流转服务中心和综合性农村产权交易平台建设；加强农村信用体系建设，稳妥推进土地承包经营权、林权抵押贷款。抓好陆良县水利建管机制和农业水价综合改革试点。

（四）抓改革促开放，增强经济发展活力

突出行政审批改革重点，支持企业创新，充分激发市场主体活力，发挥改革创新和招商引资的乘数效应。

以改革激发市场活力。做好简政放权的“加减法”，大幅减少投资项目前置审批，取消全部非行政许可审批事项，推进网上行政审批服务大厅建设，整治“红顶中介”，发挥市场在资源配置中的决定性作用。深化商事制度改革，大力培育和完善市场体系，加快建立市场主体信用信息公开系统。综合运用财政扶持、小额担保贷款和贴息等政策措施，继续抓好“两个10万元”微型企业培育工程，推动大众创业、万众创新。深化预算管理制度改革，完善预算体系，提高预算透明度和财政资金使用效益；逐步理顺市县事权与支出责任，加强政府性债务管理；落实“营改增”、资源税等税制改革措施。推进社会组织直接登记管理体制改革，扩大政府购买服务范围。做好投融资体制改革的“乘法”，推行政府和社会资本合作模式（PPP），建立PPP试点项目库，大力吸引社会资本、增加社会投资。建立健全银政、银企、证企、保企合作平台，支持企业开展直接融资，鼓励民间资本发起设立产业投资基金和股权投资基金，构建金融服务实体经济长效机制。

以创新提升竞争力。以企业为主体、市场为导向、高校院所为依托，支持企业建设科技创新平台，鼓励社会资本组建科技孵化器，积极支持院士工作站、产业技术创新联盟、企业技术中心建设。新认定国家级高新技术企业15户、省级创新型试点企业3户。引导企业加强商标、专利等知识产权的注册、使用和管理，力争新增注册商标620件。

以开放拓展发展空间。抓住国家推进“一带一路”、长江经济带、滇中城市经济圈等战略和“东产西移”重大机遇，将招商引资与产业转型升级相结合，推动曲靖综合保税区建设前期工作，打造承接东部产业转移的新高地。围绕资源禀赋、主导产业和新兴产业，推出一批招商引资项目。充分发挥产业招商组、驻外招商分局、行业协会和驻曲商会的作用，健全重大招商项目督办机制，完善招商引资组织领导、完成目标和部门考核机制，提高招商项目签约率、资金到位率、开工率和竣工（投产）率，确保实际到位市外国内资金突破800亿元，增长80%，实际利用外资突破1.3亿美元，增长30%。外贸进出口总额突破5.4亿美元，增长20%。

（五）抓好统筹协调，加快新型城镇化建设步伐

坚持科学规划、集约建设和精细管理，加快新型城镇化建设步伐，确保城镇化率达43.6%。

完善城乡规划体系。高度重视规划工作，开展“多规合一”试点，建立市县统一的规划管理信息平台。完善市域城乡规划体系，完成曲靖城市总体规划修编和市政系统综合规划、南盘江沿线区域保护利用规划、寥廓山风景名胜区规划，优化城市空间布局和形态，合理确定城市规模、开发边界、开发强度和保护空间，防止城市无序扩张。

加快珠江源大城市建设。认真对接和落实滇中城市经济圈一体化发展总体规划，启动413个、年度投资683亿元的项目建设，加快“麒沾马”同城化步伐。加大“四城联创”力度，巩固创建成果。推进中心城区人居环境提升行动，加快“五纵五横”城市道路地下管沟及地面改造提升、珠江源大道综合管廊、五大人工湖等项目建设，打通15条断头路。积极争取城市地下综合管廊、海绵城市、智慧城市建设试点。统筹布局教育、医疗卫生、文化体育、养老等公共服务设施，加快城市公交和慢行系统建设，提高城市综合承载能力。

推动县城和小城镇协调发展。加快构建中心城市、县城和小城镇合理分工、功能互补、协同发展的城镇体系。启动国家新型城镇化综合试点，深化户籍制度改革，建设城乡统筹改革信息系统和数据库，全面实行城乡统一的户口登记制度，促进农业转移人口享受城镇基本公共服务。启动35个城市棚户区改造项目建设，基本建成保障性、棚户区改造住房1.2万套，开工建设公租房2893套。强化用地保障，创新筹资方式，整合建设资金，继续抓好20个省级特色小城镇和11个市级重点小城镇建设，完善基础设施，强化产业支撑，彰显城镇个性魅力。

加强城镇管理。坚持建管并重，制定贯彻落实《云南省曲靖城市管理条例》配套办法，理顺城市管理体制机制，建立统一的城市管理基础信息平台，加强城市规划执法能力建设，探索建立及时、精细、长效的网格化管理模式。继续强化车辆乱停乱放、违规设置户外广告、搭建临时建筑等突出问题整治。

（六）抓生态优环境，争当生态文明建设排头兵

像保护眼睛一样保护生态环境，推动绿色、循环、低碳发展，提高可持续发展能力。

加大生态环境治理力度。推进三年城乡绿化攻坚行动，实施“四绿工程”，完成营造林58万亩以上。加大饮用水源地、自然保护区、湿地公园和生物多样性保护力度。实施农业面源污染综合治理，重点抓好残膜、化肥、农药、畜禽养殖等污染整治，提高农业生态安全水平。强化重金属污染防治，逐步解决历史遗留问题。坚持源头严防、过程严管、后果严惩，加强对重点区域、重点流域、重点行业、重点企业的环境管控，提高生态环境违法成本。

加大节能减排力度。在火电、水泥、钢铁等高耗能、高排放行业实施100个技改项目，淘汰落后产能73万吨以上；实行GDP能源消费总量双重调控，确保单位生产总值能耗下降3.3%。实施190个重点减排项目，严控新增污染物排放量，全面完成“十二五”节能减排目标。加快危险废物处置项目建设，实施大气污染防治行动计划，做好中心城区空气质量监测发布工作。提高城镇污水处理能力，完成89公里污水管网和88个建制镇“一水两污”项目建设。全面推广合同能源管理、污染第三方治理和节能环保产品。

推进资源节约集约利用。坚守耕地保护红线，强化基本农田保护和规划管控，科学设定优化开发、重点开发、限制开发和禁止开发区域，从源头上控制和减少占用耕地。严格执行容积率、投资强度控制指标，加强城镇土地集约利用评价。有序推进低丘缓坡土地综合开发利用试点，清理处置闲置低效利用土地，建立城镇低效用地再开发和存量建设用地退出激励约束机制。强化取水许可监督管理，巩固节水型社会试点成果。

（七）抓实事惠民生，让广大人民群众共享改革发展成果

坚持保基本、兜底线，加大社会事业和民生投入，织密织牢保障民生安全网。

推动城乡教育资源均衡配置。启动曲一中新校区和市二中改扩建等项目建设，确保市民中新校区建成投入使用，加快曲一中卓立学校建设。落实中小学、幼儿园建设补助政策，启动200所农村义务教育薄弱学校改造和29所市县中心城区中小学、26所标准化幼儿园新建工程，加快中小学信息化建设。完善优秀骨干教师市政府特殊津贴制度。开展职业教育质量提升工程，全面提高毕业生技能水平。鼓励发展民办教育和继续教育，扶持发展特殊教育。狠抓校园安全管理，打造平安和谐校园。

加强医疗卫生服务体系建设。加快市妇幼保健院、市中医院、市全科医生临床培养基地建设，新建7个乡镇卫生院、95个村（社区）卫生所（室）。推进以县级医院为重点的公立医院改革，完善基本药物制度，推广区域医疗联合体模式。继续推进乡村医生3年培养行动计划，实施基层中医药服务能力提升工程，加快县级中医院标准化和重点专科、特色专科建设。认真落实计划生育基本国策及“单独两孩”政策，确保人口自然增长率控制在6‰以内。

抓好文化体育工作。实施好文体惠民工程，推动基本公共文化服务标准化、均等化，新建25个乡（镇、街道）文化站和一批文体示范小广场。加强重点文化遗产保护、传承和利用。创新文体场馆运营模式，引入社会资本参与文体公园运营，打造高原精品赛事品牌。做好广播电视、地方志、社会科学等工作，启动市档案馆建设。

促进城乡充分就业。把扩大就业创业作为促增收的主渠道，落实鼓励创业带动就业政策，继续实施大学生创业引领计划、职工人才工程、农民工职业技能提升计划，积极做好“贷免扶补”工作，扶持1.4万人创业，新增城镇就业岗位4万个、就业人员3.2万人，城镇登记失业率控制在4.3%以内。

完善社会保障体系。扩大社会保险参保覆盖面，逐步提高社会保险待遇。落实机关事业单位养老保险改革政策。推进城镇居民医保与新农合统筹试点，提高城镇居民和新农合医保财政补助标准、个人缴费水平。完善落实社会救助和城乡低保、五保供养政策，提高优抚保障水平。新建 30个社区居家养老服务中心、10个农村敬老院，扶持发展10个养老机构，新增养老床位4200张。

抓好会泽地震灾区恢复重建。落实灾后恢复重建总体规划及实施方案，全面完成灾区民房恢复重建和修复加固任务，启动灾区教育、卫生、交通、水利等公共基础设施恢复重建。

开展民族团结进步边疆繁荣稳定创建工作，改善民族地区生产生活条件，加快民族地区经济社会发展。依法管理宗教事务，巩固发展民族团结、宗教和顺的良好局面。继续做好国防动员、双拥、外事侨务、统计调查、移民开发、保密、人防、水文等工作。支持妇女儿童、工会、共青团、红十字、慈善、残疾人等事业发展。

（八）抓安全促发展，确保经济社会健康稳定

树牢安全生产底线思维和红线意识，落实监管责任和主体责任，全力抓好安全生产。

落实安全生产责任体系。严格落实“党政同责、一岗双责、齐抓共管”的安全生产责任体系，实现市、县、乡、村“四级五覆盖”。坚定不移落实好企业主体、部门监管和属地管理责任，完善安全生产监管工作规范和监管职责清单，构建网格化监管格局，形成自上而下、职责清晰的工作机制。落实煤矿安全隐患举报奖励和驻矿监督员管理制度，全面推行政府购买第三方安全监管、企业信息公开和安全隐患分级交叉排查治理机制。

深入开展重点领域专项整治。坚持常态化安全生产专项整治，打好重点领域安全生产攻坚战。强化联合执法和“四个一律”措施，持续开展打非治违专项行动。采取“1+3”安全大检查方式，抓好煤矿、非煤矿山、危险化学品、道路交通、消防、特种设备等重点领域的隐患排查治理，坚决杜绝较大以上事故发生，确保事故起数、死亡人数、受伤人数和直接经济损失持续下降。提高防灾减灾和应急能力，推进市防灾减灾教育与调度中心建设。 强化食品药品安全监管。完善四级食品药品监管体系和生产经营准入制度，加强食品药品检验检测能力建设，深入开展食品药品安全创建活动，严厉打击食品药品违法犯罪行为，确保食品药品安全。

（九）抓法治促和谐，提高法治政府建设水平

加快建设职能科学、权责法定、执法严明、公开公正、廉洁高效、守法诚信的法治政府。

营造法治环境。坚持用法治思维、法治方式和法律手段维护公共安全，努力在全社会形成遇事找法、解决问题用法、化解矛盾靠法的良好法治环境。加强社会治安防控，健全立体化社会治安防控体系，打好禁毒防艾人民战争，依法打击邪教组织和各种违法犯罪活动，保持反恐高压态势。完善信访问题依法治理和五级联动排查化解矛盾纠纷机制，坚持属地原则与事权原则相结合，强化源头预防，畅通信访渠道，强化风险评估和舆情研判，加大矛盾纠纷排查调处力度。

坚持依法行政。制定深入推进依法行政和加快建设法治政府的实施意见，健全依法决策机制，坚持公众参与、专家论证、风险评估、合法性审查、集体讨论决定的重大行政决策法定程序。建立政府法律顾问制度，落实重大决策终身责任追究制度及责任倒查机制。自觉接受市人大的法律监督、工作监督和市政协的民主监督，认真办理人大代表议案、建议和政协委员提案。全面推进政务公开，主动接受人民群众和新闻舆论的广泛监督，让权力在阳光下运行。

加快职能转变。深化行政审批制度改革，建立权力清单、责任清单和负面清单，坚持法定职责必须为、法无授权不可为，最大限度减少政府对微观事务的管理；着力建设统一开放、竞争有序的市场体系，清除市场壁垒，建立公平开放透明的市场规则。稳步推进政府机构改革，理顺部门之间的职责关系，加快构建精干高效的政府组织体系。加快事业单位分类改革。完成行政机关和参公管理单位公务用车制度改革，提高公共资源市场化配置水平。

狠抓勤政廉政。强化督查督办和绩效考评，积极引入第三方评估，强化责任追究，确保政令畅通、决策落实、政策落地。深入开展“三严三实”和“忠诚干净担当”专题教育，在政府系统树牢人人都是发展环境、个个都是第一服务窗口的理念。进一步精简会议、文件，严禁新建楼堂馆所。严格落实“一岗双责”党风廉政建设责任制，健全廉政风险防控机制，加强对财政资金分配使用、政府性投资、政府采购、公共资源转让等重点领域和关键环节的监督制约，继续推进审计监督全覆盖，加强政府性投资项目全过程审计监督，真正形成干部清正、政府清廉、社会清淳的政治生态。

各位代表，全市人民对幸福生活的向往和追求，是我们工作的动力和努力的方向。让我们在市委的坚强领导下，恪守忠诚干净担当要求，以干在实处、走在前列的精神状态，坚定信心、凝聚力量，为创造曲靖更加美好的明天而努力奋斗！
